# Burden of neglected tropical diseases and access to medicine and diagnostics in Ethiopia: a scoping review

**DOI:** 10.1186/s13643-023-02302-5

**Published:** 2023-08-14

**Authors:** Agumasie Semahegn, Tsegahun Manyazewal, Eyerusalem Getachew, Bethelhem Fekadu, Esubalew Assefa, Munir Kassa, Gail Davey, Michael Hopkins, Mesele Araya, Tassew Woldehanna, Charlotte Hanlon, Abebaw Fekadu

**Affiliations:** 1 Centre for Innovative Drug Development and Therapeutic Trials for Africa (CDT-Africa), College of Health Sciences, Addis Ababa University, Addis Ababa, Ethiopia; 2 College of Health and Medical Sciences, Haramaya University, Harar, Ethiopia; 3 Department of Population, Family and Reproductive Health, School of Public Health, Unversity of Ghana, Accra, Ghana; 4 Health Economics and Policy Research Unit, Wolfson Institute of Population Health, Queen Mary University of London, London, UK; 5 Department of Economics, Faculty of Arts and Social Sciences, The Open University, Milton Keynes, UK; 6 Ministry of Health of Ethiopia, Addis Ababa, Ethiopia; 7 Global Health & Infection Department, Brighton and Sussex Medical School, Brighton, UK; 8 School of Public Health, College of Health Science, Addis Ababa University, Addis Ababa, Ethiopia; 9 Science Policy Research Unit, University of Sussex, Brighton, UK; 10 College of Business and Economics, Addis Ababa University, Addis Ababa, Ethiopia; 11 Policy Studies Institute, Addis Ababa, Ethiopia; 12 Centre for Global Mental Health, Health Service and Population Research Department, Institute of Psychiatry, Psychology and Neuroscience, King’s College London, London, UK; 13 Department of Psychiatry, WHO Collaborating Centre for Mental Health Research and Capacity Building, School of Medicine, College of Health Sciences, Addis Ababa University, Addis Ababa, Ethiopia

**Keywords:** Neglected tropical diseases, Diagnosis, Treatment, Medicine, Scoping review, Ethiopia

## Abstract

**Background:**

More than 1.7 billion people are affected by neglected tropical diseases (NTDs) worldwide. Forty percent of the NTD-affected people live in Africa with the poorest, most vulnerable, and hard to reach geographical areas. The NTDs cause significant social and economic burden and deepen marginalization and stigmatization. The World Health Organization’s current roadmap for NTD aims to prevent, control, eliminate, or eradicate 20 tropical diseases. Ethiopia experiences a high burden of these diseases, but current access to diagnostics, medicine, and/or care has been little explored to inform the country’s NTD strategic plan. The overall purpose of the scoping review was to map and characterize the burden of NTDs and challenges in access to diagnostics, medicine, and/or care in Ethiopia.

**Methods:**

A systematic search of evidence was conducted in PubMed, Cochrane Library, and Google Scholar from January 2000 until May 2022, without restrictions of language or study design. The Preferred Reporting Items for Systematic Reviews and Meta-Analyses Extension for Scoping Review was followed for screening of studies. Key findings were extracted and narrated qualitatively.

**Results:**

The search resulted in 4532 articles, of which 105 met the inclusion criteria and were included in the scoping review under three themes: burden of NTDs, access to diagnostics, medicine and/or care, and key barriers. Although gains have been made in the prevention and control of NTDs in Ethiopia, the burden remains high, and progress in access to diagnostics, medicine/drugs, and/or care is very slow. Poverty, poor quality of life, and underfunding of NTD programs decelerate the process of NTD elimination program in the country.

**Conclusions:**

The scoping review identified a considerable number of studies on the burden of NTDs in Ethiopia and strategies for diagnosis, treatment, and/or care; however, there is a paucity of evidence on the suitability and potential benefits of novel diagnostic technologies and medicines in the country. A regular review and analysis of such country-level evidence is important to inform the country NTDs roadmap and local implementation strategies.

**Supplementary Information:**

The online version contains supplementary material available at 10.1186/s13643-023-02302-5.

## Background

Neglected tropical diseases (NTDs) are a group of more than twenty preventable and treatable infectious diseases in the tropics [1], which affect around 1.7 billion people in 149 countries worldwide [2–4]. Of these, 40% live in Africa with the poorest and most vulnerable people living in hard to reach geographical areas [3]. NTDs cause significant social and economic burden and deepen marginalization and stigmatization [5]. NTDs are also associated with huge costs and losses to productivity [6]. The World Health Organization’s (WHO) new roadmap for NTDs (2021–2030) is looking ahead to prevent, control, eliminate, or eradicate 20 tropical diseases [7]. The roadmap is aimed at reducing morbidities and mortalities from vector-borne diseases and achieving integrated coverage of preventive chemotherapy for NTDs [2, 7]. Ending the epidemics of infectious diseases including NTDs is one of the United Nations (UN) Sustainable Development Goals (SDG:3) under target 3.3 [8]. In response to this, the WHO urged countries to develop national roadmaps on NTDs by 2020 to sustain enhanced and equitable access to high-quality healthcare coverage against NTDs by 2030. More than 74 countries [2], including Ethiopia [5, 9, 10], are ready to implement national NTDs roadmap, stimulating increased demand for program implementation and donated medicines crucial to reach the roadmap’s targets.

Almost all regions of Ethiopia have been affected by at least three NTDs. The burden is higher in the central, western, and northwestern parts of the country [11], and more than 75 million people were at risk of infection by at least one NTD in Ethiopia [12]. Over a third of those in need (27 million) had not received treatment in 2016 [3]. In response to this, increasing attention has been given to NTDs over the last couple of decades in Ethiopia [2, 5, 12]. The ambitious goals of ending NTDs have to be matched by strategies tailored to local settings, evidence-based decision-making, and sufficient access to therapeutics [13]. Likewise, there is an urgent need for effective and efficient data monitoring and national surveillance systems to enable early detection and mitigation of the spread of NTDs [7, 14].

Quality evidence is required to inform NTD policymaking and implementation Ethiopia. However, there is a paucity of synthesized evidence relating to disease burden, challenges around access to diagnostics, drugs/therapeutics, and/or care to inform NTD policymaking and program implementation in Ethiopia. The main aim of this scoping review was to map the available research undertaken on the burden of NTDs and implementation key challenges on access to diagnostic and treatment services in Ethiopia.

### Scoping review question(s)

▪ What is the scope and volume of literature on NTDs in Ethiopia?
▪ What is the burden of NTDs in Ethiopia?
▪ What are the available NTD diagnostics, medicines, and/or care for in Ethiopa?
▪ What are the challenges to NTD diagnostics, medicine, and/or care in Ethiopia?

## Methods

The methodology of this scoping review was developed based on the recommendation of Preferred Reporting Items for Systematic Review and Meta-Analyses Extension for Scoping Reviews (PRISMA-ScR) statement [15, 16], and items in the PRISMA-ScR checklist are completed [16] (Additional file 1). The type of NTDs to be considered in this scoping review was informed by the revised national NTDs master plans of Ethiopia [5, 10].

### Search and identification of studies

Studies were searched from major electronic databases that include both published and unpublished (gray literature) and available from January 2000 to May 2022. The year 2000 was selected to capture comprehensive evidence since the inception of the Millennium Development Goals (MDGs) [17, 18]. Systematic search was carried out to retrieve studies indexed in PubMed, Cochrane Library, and direct search from Google Scholar (Additional file 2). Medical Subject Headings (MeSHs) were used to search studies from the databases that were designed considering the participants, concepts, and contexts (PCC) of the research questions. Terms included NTDs prioritized in Ethiopia (onchocerciasis, trachoma, lymphatic filariasis, podoconiosis, soil transmitted helminthiasis, schistosomiasis, leishmaniasis, scabies, and dracunculiasis/guinea-worm), poverty-related disease, key challenges, barriers, associated factors, determinants, constraints, prevention and control, and Ethiopia (Table 1). Generally, MeSH terms were combined according to the databases interface compatibility and recommendation. All the searches were imported into EndNote citation manager, and duplicates were removed.

**Table 1:** Search terms using participant, concept, and context (PCC) framework

Participants	Studies conducted among adults, women, children, women-child pairs, and NTDs patients/victims
Concept	Burden/prevalence of NTDs (onchocerciasis, trachoma, dracunculiasis/guinea-worm, lymphatic filariasis, soil-transmitted helminthiasis, schistosomiasis, leishmaniasis, podoconiosis, and scabies)
	Diagnostics used to investigate onchocerciasis, trachoma, dracunculiasis/guinea-worm, lymphatic filariasis, soil-transmitted helminthiasis, schistosomiasis, leishmaniasis, podoconiosis, and scabies
	Access to treatment or care: onchocerciasis, trachoma, dracunculiasis/guinea-worm, lymphatic filariasis, soil-transmitted helminthiasis, schistosomiasis, leishmaniosis and podoconiosis, scabies
	Challenges, policy documents, NTDs roadmap
Context	Studies conducted in Ethiopia
	Studies published from January 2000 to May 2022

### Eligibility criteria

Existing evidence was included into the scoping review using the following eligibility criteria:

▪ Studies focused on prioritized NTD in Ethiopia according to the WHO definition.
▪ Any type of study — observational, interventional, evaluation study designs, and reviews
▪ Both facility- and community-based studies conducted in Ethiopia
▪ Both published and unpublished (gray literature) studies
▪ Year of publication: January 2000 to May 2022

### Selection process

**Fig. 1:**
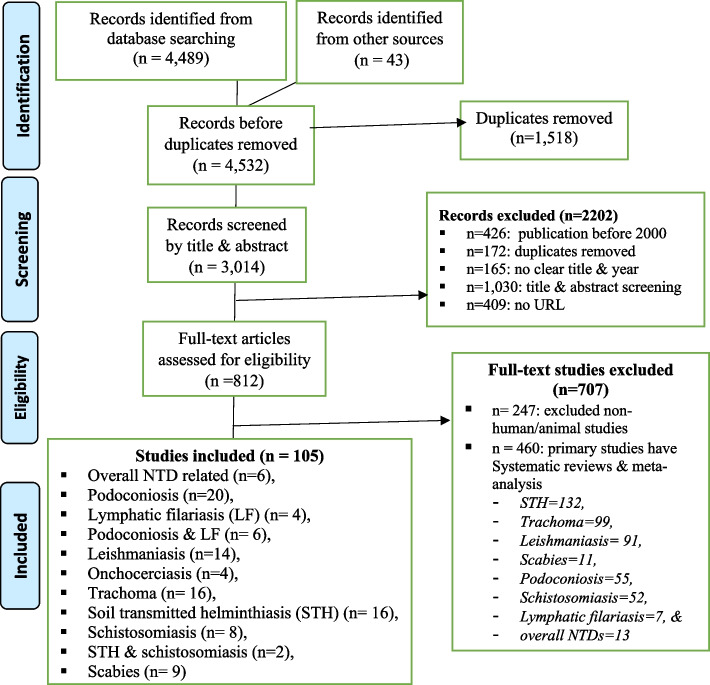
Study selection process (NTD, neglected tropical disease; LF, lymphatic filariasis; STH, soil-transmitted helminthiasis)

Studies selection was carried out in a stepwise process. Initially, screening of studies was performed based on their title and abstract by two authors (A. S. and E. G.). Studies with title and abstract that clearly stated one or more of the prioritized NTDs in Ethiopia, associated factors, access to diagnostics, medicine, and/or care were considered for further evaluation. Second, the same two authors (A. S. and E. G.) performed full-text assessment of included studies. The full-text assessment involved evaluation of the study design, sample size, participants recruitment, data analysis, presentation of key findings, conclusions, and recommendations. Third, any uncertainty or disagreement between the two full-text assessors was resolved by consensus through consultation of senior authors (A. F. and T. M.). Study screening and selection were guided by the PRISMA-ScR flowchart [15] (Fig. 1).

### Measurement of outcome and exposure

The burden of the neglected tropical diseases, access to diagnostics, medicine (drug/therapeutics), and/or care were considered as primary outcomes. The key barriers to access to diagnostic, medicine and/or care, and identified key policy recommendation to prevent and control NTDs in Ethiopia were consider as exposure.

### Data charting and summary of results

The selection process was guided by existing methodology recommendations [19]. Data charting (extraction) template was designed in Microsoft Word to record key findings of included studies (Additional file 3). The main characteristics of individual studies such as author, publication year, study area (specific geographic, administration site and regional state, national), aim of the study, design, sampling method, and type of NTDs considered in the study, burden of NTDs, diagnostic method used, medicine (drug/therapeutics) used and/or care, and other key findings were recorded in the data abstraction template. The content of the studies was grouped into three thematic areas: burden of NTDs (theme 1), studies on diagnostic (theme 2), and access to medicine (drug/therapeutic) services (theme 3).

## Results

### Description of characteristics of studies

A total of 4532 articles related to NTDs in Ethiopia were retrieved. Of these, 1518 duplicates were removed. Next, documents were screened by title, abstract, and full text to decide their eligibility for the scoping review. Finally, 105 articles were included into the scoping review. Details of the screening and selection process are depicted using a PRISMA-ScR flowchart on Fig. 1, and the detail profile of the included article is presented as an additional file (Additional file 3).

### Evidence on burden of NTDs in Ethiopia

We identified 31 articles that report the burden of prioritized NTDs in Ethiopia [3, 10, 11, 20–47]. Most were systematic reviews and meta-analyses on trachoma [20], leishmaniasis [21–25], soil-transmitted helminthiasis (STH) [26–30], schistosomiasis [30–33], scabies [34], lymphatic filariasis (LF) and podoconiosis [35–38], review on onchocerciasis [40], and historical review of the overall NTDs in Ethiopia (1941–2019) [39]. Trachoma, podoconiosis and leishmaniasis [3, 11], onchocerciasis, LF, schistosomiasis, STH, and scabies [3] are three common NTDs in Ethiopia. The government of Ethiopia has prioritized nine NTDs for intervention [10]. The STHs are common among both adults and school children in Ethiopia [26, 39, 41–44]. One-third of infants in Ethiopia are affected by STHs (specifically ascariasis) [11]. Podoconiosis and trachoma are also common in Ethiopia, mainly in Amhara, Oromia, and Southern Nation, Nationalities and Peoples (SNNP) regions [45]. The distributions of NTDs at country level have been mapped and endemic areas identified and prioritized [36, 46, 47].

#### Podoconiosis and lymphatic filariasis (LF)

Both podoconiosis and LF are among the prioritized NTDs in Ethiopia [10]. Thirteen relevant articles were identified [10, 35, 37, 48–56], which report the burden of podoconiosis and/or LF in Ethiopia [35, 52, 53]. The burden of podoconiosis is widespread in Africa which ranged from 0.1 to 8.1%, where Ethiopia registered the second highest prevalence rate (7.5%) next to Cameroon (8.1%) [10, 37, 38, 48, 50]. Podoconiosis has gained attention recently in Ethiopia after evidence emerged that it is a preventable noninfectious geochemical form of lymphedema caused by exposure of bare feet to irritant alkalic clay soil [49, 50]. The prevalence of podoconiosis in Ethiopia shows marked regional variation, as high as 8.3% in SNNP, 4.0% in Oromia, and 3.9% in Amhara region [10]. In addition, there was disparity in the burden of podoconiosis by gender where prevalence was higher among women (3.7%) than men (2.4%) [51]. Leg lymphedema has a significant negative socio-economic impact on people affected by podoconiosis and LF and their caregivers in co-endemic districts of Ethiopia [56] and also increases mortality [55].

#### Trachoma

Five studies were identified on the burden of trachoma in Ethiopia [20, 57–60]. The overall prevalence of active trachoma among children in Ethiopia was 26.9%, and was highest in SNNP (35.8%) followed by 30.2% in Amhara and 20.2% in Oromia [20]. Studies conducted in Amhara region (152–160) have shown that 28% of districts had trachoma, with prevalence ranging from 8.5 to 36% [57, 58]. A high burden of trachoma was also reported in studies conducted in Wolayita Zone [59, 60].

#### Soil-transmitted helminthiasis (STH)

Systematic reviews and meta-analyses identified STH burden [27–30, 41, 43, 61, 62]. In five studies, the prevalence of STH in Ethiopia ranged from 13 to 52.4% [27–30, 39, 41]. Of these, *Ascaris lumbricoides* (8.8 to 14.0%) [27, 28, 30, 41, 43], hookworm (9.5 to 12.5%) [28–30, 41], *Trichuris trichiura* [30, 41], and *Strongyloides stercoralis* (5.6%) [29] were the most common STHs in Ethiopia. The burden of parasite among pregnant women was 29% in Ethiopia [43]. Furthermore, the prevalence of *Strongyloides stercoralis* in Ethiopia ranged from 1.8 to 11.1% in adults [61, 62], from 0.3 to 20.7% in children, and 1.5 to 17.3% in HIV-positive adults [62].

#### Schistosomiasis

Overall, the prevalence of schistosomiasis varies across regions of Ethiopia from 39.8 to 41.5% in SNNP [31, 32], 41% in Amhara, 31.4% in Tigray, 28.9% in Oromia [32], and 15% in Afar [31]. In four studies, the prevalence of schistosomiasis was as high as 73.9% in some districts of Ethiopia and with lower figures 2% [31–33, 39]. Among school-age children, males were 58% more likely to be infected with schistosomiasis than females [31]. Hepatosplenic schistosomiasis is another ignored morbidity in Ethiopia [63].

#### Leishmaniasis

Leishmaniasis is another prioritized NTD with a report of high morbidity and mortality in Ethiopia [10]. Five studies reported the prevalence of leishmaniasis [21–25]. The prevalence of leishmaniasis in high burden areas of Ethiopia was between 9.1% and 19% [21, 25]. The prevalence of leishmaniasis was 39.1% in Amhara region, followed by 23% in Tigray region [21]. Visceral leishmaniasis was one of the common HIV co-infections (5.2%) [24]. Being male was associated with an increased risk of leishmaniasis diagnosis [21].

#### Scabies

The prevalence of scabies is documented in several studies in Ethiopia [34, 64–67]. In one systematic review, the overall prevalence of scabies infestation in Ethiopia was 14.5% [34]. Scabies was more common among people who have frequent contact with people with active scabies at home, who do not use soap/detergent for hand-washing, and move from non-endemic to endemic areas [68]. Scabies has a strong relationship with malnutrition among orphan children in Ethiopia [69].

#### Onchocerciasis

Onchocerciasis has been known in Ethiopia since 1939 and listed as a priority NTD in 2013 [9, 10, 70]. Although onchocerciasis affects millions of people in wide geographic area, and the transmission of onchocerciasis in many districts has remained persistent, Ethiopia envisaged to interrupt onchocerciasis transmission by 2020 and to be certified free from onchocerciasis by 2025 [40]. In one study, the mean microfilaremia (mf) of onchocerciasis in Ethiopia was 4.7% (village range 1.1–11.0%) [71].

### Evidence on access to diagnostics and its challenges

#### Leishmaniasis

Peripheral blood microscopy [72], kinetoplast deoxyribonucleic acid (kDNA) polymerase chain reaction (PCR) method [73], quantitative real-time kinetoplast deoxyribonucleic acid (qRT-kDNA) PCR [74], diagnosis of leishmaniasis based on rK39 immunochromatographic (rK39-ICT) [75, 76], in-house liquid direct agglutination test (AQ-DAT) antigen [77], microculture method (MCM), traditional culture method (TCM) and smear microscopy [78], and molecular diagnosis [25] are available diagnostic procedures for leishmaniasis in Ethiopia. Visceral leishmaniasis can be ruled in with peripheral blood microscopy in a substantial number of suspected cases and may reduce the number of tissue aspirations performed. However, more sensitive and logistically feasible methods than light microscopy are needed to detect the parasites in the blood [72]. The kDNA PCRs showed excellent performance for diagnosis of *Leishmania aethiopica*. The dry blood sample (DBS) can be used for PCR in microscopy is negative, where kDNA PCR method is available [73].

The qRT-kDNA PCR is a highly sensitive test [74], but the sensitivity of rK39-ICT is low, and its specificity is poor in Ethiopia as compared with splenic aspiration as a gold standard test. So the rK39-ICT needs improvement for clinical use for visceral leishmaniasis in Ethiopia [75, 76]. In-house AQ-DAT is an accessible diagnostic method that minimizes intermittent stock outs and could strengthen the national visceral leishmaniasis control program [77]. In addition, the microculture method (MCM) is a more sensitive and rapid culturing method for the isolation of *Leishmania aethiopica* than traditional culture method (TCM) and smear microscopy [78]. The molecular diagnostic method has significantly lower prevalence than microscopic examination [25].

#### Trachoma

In addition to clinical evaluation, three studies reported diagnostic methods for trachoma [79–81]. Photo evaluation (standard 3D images) is the chief diagnostic approach for trachomatous trichiasis (TT) [79], while Chlamydia trachomatis deoxyribonucleic acid (DNA) [80] and putative attractants [81] were the diagnostic methods for active trachoma. Trachoma can also be detected using Chlamydia trachomatis DNA on the hands, faces, or clothing of the individuals living with ocular-positive household members [80]. In addition, putative attractants may play a role in identifying short-chain fatty acids and aromatic compounds that are detected by the antennae of *M. sorbens*. Further work is required to optimize chemical blends and release rates, to produce a synthetic lure to which the behavioral responses of *M. sorbens* can be investigated [81].

#### Soil-transmitted helminthiasis (STH)

Combinations of formol-ether concentration, Baermann concentration, and molecular methods, Kato-Katz, direct saline microscopy, and formol ether concentration methods [61] are available in Ethiopia. Kato-Katz, McMaster, and Mini Parasep® SF were used to diagnose schistosomiasis [82], while quantitative polymerase chain reaction (qPCR) [83], serology, and PCR are diagnostic test for strongyloidiasis [62]. Kato-Katz thick smear, Kato-Katz thick smear and formol-ether concentration, and triple urine-circulating cathodic antigen (CCA)-cassette are the diagnostic test for *S. mansoni* [32]. Direct wet mount microscopy (DWMM) Kato-Katz, McMaster, and Mini-FLOTAC are used to diagnose both trichuris and hookworms [84].

The sensitivity of Mini Parasep® SF, Kato-Katz, and McMaster tests for detecting at least one species of parasites was 90.2%, 62.4%, and 80.0%, respectively. The specificity of these tests was 44.5%, 55.2%, and 26.5%, respectively. The Mini Parasep® SF fecal parasite concentrator technique showed better performance than Kato-Katz and McMaster techniques in detecting STHs in stool samples, particularly for *S. mansoni* and *A. lumbricoides*. Hence, Mini Parasep® SF could be used as one of the suitable fecal examination methods for surveillance, monitoring, and evaluation of preventive chemotherapy of schistosomiasis [82].

Likewise, qPCR is the method used in the monitoring and evaluation to confirm cessation of program [83]. Serology and PCR diagnostic tests have four times the capacity for diagnostics of strongyloidiasis than microscopy techniques [62]. Kato-Katz thick smear; Kato-Katz thick smear and formol-ether concentration, and triple urine-CCA cassette are the commonly used diagnostic methods for *S. mansoni* among children in Ethiopia [32]. The diagnostic sensitivity of DWMM was compared to a composite reference standard (CRS) consisting of Kato-Katz, McMaster, and Mini-FLOTAC for the diagnosis of STHs. The sensitivity of DWMM was 73.8% for *Ascaris* but was around 17% for both trichuris and hookworms [84].

#### Podoconiosis and LF

Clinical history, physical examination, and tests to rule out other forms of lymphedema together make up the important diagnosis algorithm that has been validated for identification of podoconiosis [85]. In addition, a new tool (3D imaging) for mapping LF was piloted in Ethiopia and found to benefit national LF programs by confirming where LF is endemic, therefore saving time and resources by preventing mass drug administration (MDA) where there is no evidence of ongoing LF transmission [86].

### Evidence on access to medicine and care for NTDs in Ethiopia

#### Leishmaniasis

Available evidence shows that sodium stibogluconate (SSG), liposomal-amphotericin B (L-AMB), a combination of SSG with paromomycin [22], antimonials with paromomycin in combination or pentamidine [87], and a combination of AmBisome and miltefosine [88] were available treatments for leishmaniasis. The overall treatment success rate was 82.6%. The treatment success rates using SSG were 81.5%, that of multiple doses of liposomal-amphotericin B (L-AMB) was 96.7%, and the combination of SSG with paromomycin (90.1%) [22]. Antimonials with paromomycin in combination or pentamidine are an effective treatment options for diffused cutaneous leishmaniasis [87]. In addition, the combination of AmBisome and miltefosine is effective strategy to treat visceral leishmaniasis in HIV-co-infected patients in Ethiopia [88].

#### Trachoma

A blended approach of latrine promotion and MDA with azithromycin has been proven to prevent trachoma in Ethiopia [89]. Mass drug administrations of azithromycin are the predominant approach to prevent trachoma in Ethiopia [90–93]. Other intervention includes doxycycline for postoperative trichiasis cases [94], TT surgery [94–96], and hygiene measures including facial cleanliness and environmental improvement (SAFE) [57]. The overall MDA coverage of azithromycin ranged from 79.5 to 93.3% in Ethiopia, which is higher than the minimum WHO set criteria of 80% [91, 92]. Misconceptions and poor mobilization were common challenges [91].

#### Soil-transmitted helminthiasis (STH)

Chemotherapy using mebendazole [97, 98] and MDA campaigns [92, 99] are used to prevent and treat STHs. Chemotherapy against STH is crucial, and its coverage was 71% in Ethiopia [97]. MDA mobilization and awareness creation campaigns targeted head of household, those in poorer health, and older age groups [92]. Access to water sanitation and hygiene (WASH) program is an important public health intervention for STHs [27, 99]. Access to WASH reduced infestation of parasites by 54% [27].

#### Schistosomiasis

Overall treatment coverage of praziquantel against schistosomiasis was 75.5% in Ethiopia [100]. Praziquantel was a drug of choice for the treatment for *Schistosoma mansoni* in Ethiopia [100–102].

#### Podoconiosis and LF care

In terms of prevention, foot hygiene in areas of irritant soil is [49]. Shoe-wearing norms are progressively changing due to secular change in Ethiopia [103]. Ivermectin MDA coverage for LF was 81.5% which is higher than the minimum recommended level of coverage (65%) [104]. Nevertheless, 75 Woredas were newly identified endemic for LF, and only 3.4% of the LF patients had received treatment [52]. A simple and inexpensive package of lymphedema self-care comprising information about foot hygiene, skin care, bandaging, exercises to improve lymph drainage, and use of socks and shoes has shown effect to reduce occurrence of acute attacks [105]. Minor surgical intervention (nodulectomy) [49, 105–107], foot hygiene and footwear [37, 49, 103, 108, 109], economic empowerment and life skill programs [49, 53, 107–112], transformation of rigid inequity gender norms [110, 111, 113, 114], awareness creation, and psychosocial support for stigma minimization [53, 109, 111, 113, 115–117] were crucial component of care for people affected by podoconiosis and LF. Several articles have documented the integrated morbidity management for podoconiosis and LF [49, 104, 106, 108, 109, 118]. Family-based intervention had played a key role in preventing impairments and reducing stigma through self-management of disabilities and improving family quality of life [112]. Among 363 health facilities surveyed, podoconiosis and LF were the major causes of lymphedema in Ethiopia, but only 24% of LF and 12% of podoconiosis patients had received care from the health facilities [106].

#### Onchocerciasis

Onchocerciasis is targeted for elimination, and the coverage of community-directed treatment (ivermectin) coverage of onchocerciasis has been around 80% and up to 85.9% in some districts in Ethiopia [40, 119–122]. Vector control was also an important prevention and control intervention [40].

### Challenges in access to medicine and care in Ethiopia

Lack of adequate resource for drug discovery, and/or low-purchasing capacity, were common challenge for access to medicine for leishmaniasis treatment in Ethiopia [123]. These were compounded by very poor access to diagnosis and, consequently, significantly delayed access to treatment for visceral leishmaniasis [124]. Similarly, the podoconiosis elimination program has been affected by lack of necessities including footwear and health education [109]. Social stigma has an immense impact on podoconiosis care and support in Ethiopia [114]. The MDA with ivermectin monotherapy did not interrupt LF transmission, but adding albendazole and improving treatment coverage are recommended to improve LF prevention [44, 71].

Gender inequity has significant impact on women’s healthcare seeking and access to medicine and/or care in Ethiopia [113]. In addition, stigmatized attitudes towards patients with podoconiosis influence patients’ seeking care. More than half (52.7%) of youths had stigmatizing attitudes towards patients with podoconiosis. Of these, 59.3% of them were female [117]. Furthermore, social and financial pressures placed on podoconiosis cases affected families and caregivers [111].

MDA intervention has been used to control scabies for many years [122]. Scabies MDA that recently employed in response to a massive outbreak in Amhara region was affected by failure to follow-up, shortage of medicine, and lack of leadership effective prioritization [122]. Likewise, MDA program coverage for onchocerciasis was significantly high among in-school adolescents with treatment offering and swallowing status [119]. Hence, school absenteeism was the main reason for not being offered ivermectin (40.9%) and not knowing about MDA (25.3%) [119].

### Strategies to improve access to medicine

The national plan for NTD prevention and control has been put forward through three consecutive national strategic documents or master plans, the first covering 2013–2015, second 2016–2020, third roadmap 2021–2025 [9, 10, 70], and NTD elimination district-level coordination toolkit [12]. Comprehensive prevention strategies, promotion of footwear, and personal hygiene are highly needed interventions [37]. Raising community awareness about NTDs, particularly podoconiosis and LF, to transform inequitable gender norms is vital to improve access to healthcare [113, 116]. Integration of the care package into routine healthcare in Ethiopia may be effective in improving health-related quality of life and disability and reducing time out of economic activity due to podoconiosis and LF [115]. Sustainable awareness creation will have a crucial contribution to minimize stigma and make patients resilient through psychosocial and life skill interventions [53]. Similarly, community engagement is essential in the success of MDA program, alongside strong political commitment, and guideline adherence [122]. In one systematic review, vaccine development was identified as an emerging science for the prevention of schistosomiasis, but this has not yet been realized. A vaccination strategy would be an ideal tool for a significant and sustainable reduction in the transmission and disease burden of schistosomiasis [125].

## Discussion

This scoping review mapped the volume of available evidence on the burden of the priority NTDs in Ethiopia, access to diagnostic, and treatment gaps. It covered studies undertaken on burden, access to diagnostic, medicine/drug and/or care for NTDs, and key program implementation challenges in Ethiopia. We found systematic reviews, geospatial mapping, national surveys, scoping reviews, primary studies, and national strategic plan or roadmaps on NTDs. The geographical coverage of NTD prevention and control has increased over time [44], with bolder strategies. Nevertheless, the evidence suggests that, despite high burden of NTDs in Ethiopia, the implementation of prevention and control programs still lags.

This scoping review was guided by the prioritized NTDs in the three master plans of Ethiopia [5, 9, 10]. Other evidence has documented that up-to-date diagnostic data is highly valuable for programmatic decision and reframing of diagnostic methods. Nonetheless, there is limited availability of diagnostic tool for many NTDs. Therefore, creation guiding framework is critical to stimulate investment on research and diagnostic support for NTDs [13]. Strong private–public partnerships, donation by pharmaceutical companies, and increased budget allocation for the NTD program are very crucial to improve access chemotherapies to eliminate NTD [3]. Nevertheless, lack of service integration and WASH are still the challenges of NTD prevention and control programs [44]. The fact that strengthening stakeholder engagement, service integration and coordination of evidence-informed interventions are crucial strategies to synergize NTD elimination programs in Ethiopia [44].

In addition, integrated NTD control, mapping, rapid scale-up of interventions, and shifting from operational research into implementation of intervention packages are very crucial to eliminate NTDs in Ethiopia [11]. Interventions such as deworming for school children, access to improved personal and environmental hygiene in school [99], community mobilization and uniform training campaign for trachoma prevention and control [91], poverty reduction to attitude transformation to minimize stigma and discriminations, and mental distress among podoconiosis-affected people [109] should be emphasized to interrupt disease transmissions and improve quality of life of NTD-affected people.

We used comprehensive evidence, both published and unpublished literature including policy documents related to NTD burden, access to medicine, and its barriers in Ethiopia, which is the strength of this scoping review. This scoping review considers all NTDs prioritized by the government of Ethiopia that made the findings very broad which is the known weakness of scoping review. Therefore, this scoping review contributes to inform the NTD elimination global policy in the Sustainable Development Goals by 2030 [126, 127] and evidence uptake for NTD policy and program design in Ethiopia. Furthermore, the findings from this scoping review are crucial to organize stakeholder consultative meetings to put a foundation for evidence to health policymaking translation platform and suggest future health care, health policy research direction, and evidence to policy strategies in Ethiopia.

## Conclusions

The scoping review has found substantial level of evidence to inform neglected tropical diseases policymaking and practice in Ethiopia. Articles identified have shown the burden of NTDs, diagnostic approaches, and treatment coverage in Ethiopia. There is relatively strong evidence on podoconiosis, trachoma, soil-transmitted helminthiasis, and leishmaniasis but much less on scabies, guinea-worm, and onchocerciasis. It is vital that available research findings are taken up to inform policymaking and practice. Although some primary studies reported diagnostic methods for the NTDs, there is little high-quality evidence on the sensitivity and specificity of these diagnostic methods. Similarly, preventive chemotherapy coverage of mass drug administration is widespread, but access to medicine and/or care for intensive diseases management is very limited. Access to diagnostics, medicine, and/or care has been affected by gender inequality, attitude towards footwear, stigma and discrimination, and economic status of the most vulnerable group of population. Evidence on access to timely and proven diagnostics, medicine, and/or care for NTD is very scarce in Ethiopia. We suggest that available evidence from nationwide surveys, mapping exercise, and systematic review and meta-analyses should be regularly reviewed and used to inform policymaking and implementation strategies or practice in Ethiopia in the future.

### Supplementary Information

**Additional file 1.** Preferred Reporting Items for Systematic reviews and Meta-Analyses extension for Scoping Reviews (PRISMA-ScR) Checklist.

**Additional file 2.** Search strategy.

**Additional file 3: Table 1.** Description of relevant evidence included in scoping review of neglected tropical diseases (NTDs) burden in Ethiopia (Additional file 3).

## Data Availability

We will make data available once completed the main scoping review and upon request to the corresponding author.

## Abbreviations

AQ-DAT: In-house liquid direct agglutination test
CCA: Circulating cathodic antigen
CDT-Africa: Centre for Innovative Drug Development and Therapeutic Trials for Africa
DWMM: Direct wet mount microscopy
kDNA: Kinetoplast deoxyribonucleic acid
LF: Lymphatic filariasis
MDA: Mass drug administration
MDG: Millennium Development Goal
NTDs: Neglected tropical diseases
PCR: Polymerase chain reaction
qPCR: Quantitative polymerase chain reaction
qRT-kDNA: Quantitative real-time kinetoplast deoxyribonucleic acid
PRISMA-ScR: Preferred Reporting Items for Systematic Review and Meta-Analysis extension for Scoping Review
rK39-ICT: RK39 immunochromatographic
SDG: Sustainable development goal
SNNP: Southern Nation, Nationality and People
STH: Soil-transmitted helminthiasis
SSG: Sodium stibogluconate
WHO: World Health Organization
