# Supplementary material for: Burden of neglected tropical diseases and access to medicine and diagnostics in Ethiopia: a scoping review
Source: Syst Rev. 2023 Aug 14;12:140. doi: 10.1186/s13643-023-02302-5 (PMC10424375; doi:10.1186/s13643-023-02302-5)
Supplement: Supplementary file 2 — Additional file 2. Search strategy. [file 13643_2023_2302_MOESM2_ESM.docx]

Search string for Scoping review on NTDs

1. PubMed database

“Neglected tropical disease” OR “neglected disease” OR NTD

OR Trachoma OR “eye infection” OR “eye disease” OR “bacterial conjunctivitis” OR “Chlamydia Infections” OR “Corneal Diseases” OR “Chlamydia trachomatis” OR “trichiasis” OR “trachomatous trichiasis” OR "Trachoma"[Mesh]

OR

Leismaniasis OR Leishmaniosis OR leishmaniases OR "leishmaniasis"[MeSH Terms] OR Cutaneous[All Fields] OR ("visceral leishmani?sis"[MeSH Terms] ^[[1]](#endnote-1)^

OR

Onchocerciasis OR Onchocercias?s OR “river blindness” OR “onchocerciasis ocular” “onchodermatitis” OR "onchocerciasis"[MeSH Terms]

OR

Podoconios?s OR “non-filarial elephantiasis” OR elephantias?s OR “lymphatic filariasis” OR OR “Bigfoot Disease* ” OR Lymphedema OR podocon* OR "elephantiasis"[MeSH Terms] OR podoconiosis[Text Word]

OR “Guinea Worm Disease*” OR “Guinea Worm” OR “Guinea Worm infection” OR Dracunculus OR Dracunculos?s OR "dracunculiasis"[MeSH Terms] OR dracunculiasis[Text Word] OR “Parasitic Diseases” OR “Helminthiasis” OR “Nematode Infections”OR “Secernentea Infections” OR “Spirurida Infections”

OR Scabies OR “Ectoparasitic Infestations” OR “[Mite Infestations](https://www.ncbi.nlm.nih.gov/mesh/68008924)” OR “Sarcoptes scabiei” OR "scabies"[MeSH Terms]

OR Schistosomias?s OR Bilharzias?s OR “Katayama Fever” OR “schistomasis Infection” OR Neuroschistosomiasis OR “Schistosomiasis haematobia” OR “Schistosomiasis japonica”OR “[Schistosomiasis mansoni](https://www.ncbi.nlm.nih.gov/mesh/68012555)”OR "schistosomiasis"[MeSH Terms]

OR Helminthiasis OR “worm infection” OR “soil transmitted helminthiasis” OR “parasitic intestinal helminths” OR tapeworms OR flukes, and roundworms OR ascarias?s OR Nematodes (roundworms) OR Cestodes (tapeworms) OR Trematodes (flukes) OR “Ascaris lumbricoides” OR Ascaris OR Ascariasis OR hookworm OR whipworm OR “soil-transmitted helminths” OR STH OR Diphyllobothriasis OR Echinococcosis OR Hymenolepiasis OR Monieziasis OR Taeniasis OR Dictyocaulus Infections OR Dirofilariasis OR Fascioloidiasis OR Monieziasis OR [Setariasis](https://www.ncbi.nlm.nih.gov/mesh/68012719) OR [Strongyle Infections OR Equine](https://www.ncbi.nlm.nih.gov/mesh/68013319) OR Toxocariasis OR Nematode Infections OR Adenophorea Infections OR Larva Migrans OR Secernentea Infections OR [Clonorchiasis](https://www.ncbi.nlm.nih.gov/mesh/68003003) OR Dicrocoeliasis OR Echinostomiasis OR Fascioliasis OR Fascioloidiasis OR Opisthorchiasis OR Paragonimiasis OR Schistosomiasis OR "helminthiasis"[MeSH Terms]

AND

Ethiopia

NB: Searches should be done by combining concept with context (Concept OR concept) and context that is Ethiopia) to get more studies comprehensively. Searching one by one will give much more studies than putting in once.

| Overall NTD | “Neglected tropical disease” OR “neglected disease” OR NTD  AND  Ethiopia | PubMed=99=119  Cochrane library: 7  Google Scholar=134 |
| --- | --- | --- |
| Trachoma | Trachoma OR “eye infection” OR “eye disease” OR “bacterial conjunctivitis” OR “[Chlamydia Infections](https://www.ncbi.nlm.nih.gov/mesh/68002690)” OR “[Corneal Diseases](https://www.ncbi.nlm.nih.gov/mesh/68003316)” OR “Chlamydia trachomatis” OR “trichiasis” OR “trachomatous trichiasis” OR "Trachoma"[Mesh]  AND  Ethiopia | PUBMED =235=295  Cochrane library:75/70  (Embase index journals not accessed)  (Trachoma in Ethiopia)  Google Scholar=212 |
| Leishmaniasis | Leismaniasis OR Leishmaniosis OR leishmaniases OR "leishmaniasis"[MeSH Terms] OR Cutaneous[All Fields] OR ("visceral leishmani?sis"[MeSH Terms]  AND  Ethiopia | Pubmed= 262=324  Cochrane library: 1  Google Scholar= 237 |
| Onchocerciasis | Onchocerciasis OR Onchocerciasis OR “river blindness” OR “onchocerciasis ocular” “onchodermatitis” OR "onchocerciasis"[MeSH Terms]  AND  Ethiopia | PubMed= 27=37  Cochrane library: 0  Google Scholar=86 |
| Podoconiosis | Podoconios?s OR “non-filarial elephantiasis” OR elephantias?s OR “lymphatic filariasis” OR OR “Bigfoot Disease* ” OR Lymphedema OR podocon* OR "elephantiasis"[MeSH Terms] OR podoconiosis[Text Word]  AND  Ethiopia | PubMed=131=160  Cochrane library: 8/15  Google Scholar=168 |
| Guindea Worm | “Guinea Worm Disease*” OR “Guinea Worm” OR “Guinea Worm infection” OR Dracunculus OR Dracunculos?s OR "dracunculiasis"[MeSH Terms] OR dracunculiasis[Text Word] OR “Parasitic Diseases” OR “Helminthiasis” OR “Nematode Infections”OR “Secernentea Infections” OR “Spirurida Infections”  AND  Ethiopia | Pubmed= 243=291  Cochrane library: 0  Google Scholar=57 |
| Scabies | Scabies OR “Ectoparasitic Infestations” OR “[Mite Infestations](https://www.ncbi.nlm.nih.gov/mesh/68008924)” OR “Sarcoptes scabiei” OR "scabies"[MeSH Terms]  AND  Ethiopia | Pubmed=51=53  Cochrane library: 0  Google Scholar=39 |
| Schistosomiasis | Schistosomias?s OR Bilharzias?s OR “Katayama Fever” OR “schistomasis Infection” OR Neuroschistosomiasis OR “Schistosomiasis haematobia” OR “Schistosomiasis japonica”OR “[Schistosomiasis mansoni](https://www.ncbi.nlm.nih.gov/mesh/68012555)”OR "schistosomiasis"[MeSH Terms]  AND  Ethiopia | PubMed= 316=349  Cochrane library: 0  Google Scholar=340 |
| STH | Helminthiasis OR “worm infection” OR “soil transmitted helminthiasis” OR “parasitic intestinal helminths” OR tapeworms OR flukes, and roundworms OR ascarias?s OR Nematodes (roundworms) OR Cestodes (tapeworms) OR Trematodes (flukes) OR “Ascaris lumbricoides” OR Ascaris OR Ascariasis OR hookworm OR whipworm OR “soil-transmitted helminths” OR STH OR Diphyllobothriasis OR Echinococcosis OR Hymenolepiasis OR Monieziasis OR Taeniasis OR Dictyocaulus Infections OR Dirofilariasis OR Fascioloidiasis OR Monieziasis OR [Setariasis](https://www.ncbi.nlm.nih.gov/mesh/68012719) OR [Strongyle Infections OR Equine](https://www.ncbi.nlm.nih.gov/mesh/68013319) OR Toxocariasis OR Nematode Infections OR Adenophorea Infections OR Larva Migrans OR Secernentea Infections OR [Clonorchiasis](https://www.ncbi.nlm.nih.gov/mesh/68003003) OR Dicrocoeliasis OR Echinostomiasis OR Fascioliasis OR Fascioloidiasis OR Opisthorchiasis OR Paragonimiasis OR Schistosomiasis OR "helminthiasis"[MeSH Terms]  AND  Ethiopia | PubMed:1076=1191  Cochrane library:6/11(1 review and 10 Trials)  Google Scholar=278 |
|  |  | Overall PubMed=2830  Overall Cochrane=91  Google Scholar=1553 |
|  | Grand total | 4489 |

1. [↑](#endnote-ref-1)
