# Supplementary material for: Burden of neglected tropical diseases and access to medicine and diagnostics in Ethiopia: a scoping review
Source: Syst Rev. 2023 Aug 14;12:140. doi: 10.1186/s13643-023-02302-5 (PMC10424375; doi:10.1186/s13643-023-02302-5)
Supplement: Supplementary file 3 — Additional file 3: Table 1. Description of relevant evidence included in scoping review of neglected tropical diseases (NTDs) burden in Ethiopia (Additional file 3). [file 13643_2023_2302_MOESM3_ESM.docx]

Table 1: Description of relevant evidence included in scoping review of neglected tropical diseases (NTDs) burden in Ethiopia (*Additional file 3*)

| **Author, year** | **Aim** | **Scope of study** | **Study design** | **Type of NTDs** | **Key findings** |
| --- | --- | --- | --- | --- | --- |
| FMOH, 2021 | Third National Strategic Plan | National | National | Prioritized NTDs | The Third National roadmap for NTDs (2021-2025): Leprosy and Rabies are included |
| FMoH, 2016 | Second National Strategic Plan | National | National | Prioritized NTDs | The national masterplan/roadmap for NTDs (2016-2020) |
| FMoH, 2013 | First National Strategic Plan | National | National | Prioritized NTDs | The national masterplan/roadmap for neglected tropical disease (2013-2015) |
| FMoH, 2018 | NTD Woreda Transformation | National | Strategic Plan | Prioritized NTDs | The national NTD woreda transformation policy directives |
| Tilahun et al., 2021 | Mapping the role of digital health technologies in the case detection, management, and treatment outcomes of NTDs | National | Scoping review | Prioritized NTDs | Use of digital health technologies is an emerging and promising way to improve disease prevention, diagnosis, case detection, treatment delivery, and patient follow-up and facilitating health facility appointments thereby improving health outcomes |
| Deribe et al., 2017 | To describe implementation of the integrated approach into the Ethiopian health system | National | Integrated service mapping | Lymphatic Filariasis (LF) & podoconiosis | An integrated approach for LF and podoconiosis morbidity management was feasible and applicable. |
| Deribe et al., 2015 | mapping and modelling the geographical distribution and environmental limits | National | Mapping and modelling | Podoconiosis | Patient having podoconiosis occurrence increased with high altitude, precipitation and silt fraction of clay soil. |
| Deribe et al., 2018 | To define the epidemiology and distribution of podoconiosis | Ethiopia/  Global | Systematic review | Podoconiosis | Podoconiosis prevalence ranged from 0.1% to 8.1% in the African region, and the prevalence of podoconiosis in Ethiopia was 7.5%. |
| Deribe et al., 2013 | Ten years of podoconiosis research in Ethiopia | National | Review | Podoconiosis | Historical profile and perspectives of the ten years progress in Ethiopia |
| Berhe et al., 2021 | To assess the effect of sex differences and pooled prevalence of podoconiosis in Ethiopia. | National | Systematic review and meta-analysis | Podoconiosis | Prevalence of podoconiosis in Ethiopia was 6% which is high, and rural resident females are at higher risk of developing podoconiosis than males. |
| Ngowi et al, 2020 | To determine the prevalence and pattern of waterborne parasitic infections in eastern Africa | Ethiopia was part of study | Systematic review | STH and schistosomiasis | Schistosomiasis prevalence ranged from 1.9% to 73.9%, and,  STH prevalence was ranging from 41.7 to 52.4%. Ascaris lumbricoides, Trichuris Trichiura and hookworms were the most common helminth |
| Hailegebriel et al., 2020 | To estimate the overall prevalence of STH at the national level and to identify high-risk regions requiring interventions | National | Systematic review and meta-analysis | STHs | Prevalence of STH was 33.4% in Ethiopia, such ascaris lumbricoides (19.9%), Trichuris trichiura (12.4%), and hookworm (7.9%). High prevalence of STH was observed in Oromia (42.5%) followed by SNNPR (38.3%) and Amhara (32.9%) regional states. |
| Chelkaba et al., 2020 | To systematically review and determine pooled prevalence of STH among children in Ethiopia | National | Systematic review and meta-analysis | STHs | Prevalence of STHs was 33% in Ethiopia, of these, 44% in SNNPR, 34% in Amhara, 31% in Oromia and 10% in Tigray regions. STHs infection rate has been decreasing from 44% pre-mass drug administration (MDA) era (1997-2012) to 30% at post-MDA (2013-2020). |
| Maddren et al.,2021 | To assess progress made by the national STH and schistosomiasis control programme | National | Systematic review and meta-analysis | STH and schistosomiasis | The prevalence of STH and schistosomiasis in Ethiopia has decreased over time due to the strategic use of anthelmintics. |
| Chelkeba et al., 2021 | to provide pooled prevalence estimate, prevalence in different regions and species-specific IPIs among pregnant women in Ethiopia | National | Systematic review and meta-analysis | Intestinal parasite | An overall prevalence of parasite infestation among pregnant women was 29%. Of these, the prevalence of IPs was 36% in Oromia, 29% in Amhara, 24% in SNNP, 24% in Tigray and 24% in Addis Ababa. By specific parasite, hookworms: 10% and Ascaris lumbricoides 10%. |
| Boltena et al., 2021 | To determine the pooled prevalence of malaria and STH coinfections among malaria suspected patients in Ethiopia | National | Systematic review and meta-analysis | STHs | The pooled prevalence of STHs co-infection with malaria was 13% in Ethiopia. The most STH detected were hookworm, Ascaris *lumbricoides*, and Tirchuris *trichiura*. |
| Worku, 2017 | To give brief highlight on NTDs program in Ethiopia, progress and challenges | National | Review | Prioritized NTDs in Ethiopia | NTDs program in Ethiopia, progress and challenges |
| Cremers et al.,2020 | To explore ethnographic barriers in tackling podoconiosis | Wolayita, SNNP | Ethnographic study | Podoconiosis | understanding of patients’ daily experiences with podoconiosis has potential impact on care, awareness and medical teaching programs. |
| Kelemework et al., 2016 | To explore barriers to footwear use and non-use in northern Ethiopia | Gojam, Amhara | Qualitative study | Podoconiosis  (Access to medicine/care) | Podoconiosis care affected by individual, cultural and socioeconomic barriers that influence victims’ decisions about and use of footwear in rural northern Ethiopia. |
| Yimer et al., 2015 | To know the epidemiology and disease burden, | National | Review | LF and podoconiosis | Disease distribution and people’s knowledge about LF & podoconiosis are not well known in Ethiopia, and data in the country are limited. |
| Rebollo et al., 2015 | To perform a nationwide mapping exercise to determine the number of people targeted for MDA | National | National mapping | LF | People living in LF endemic areas is 60% lower. Integrated mapping of multiple NTDs is feasible and cost effective and if properly planned, can be quickly achieved at national scale. |
| Davey et al., 2007 | To assess the history, epidemiology, genetics, ecology, pathogenesis, pathology and management of podoconiosis | National | Review | Podoconiosis | Podoconiosis is unique in being an entirely preventable non-communicable disease. Podoconiosis develops in men and women working barefoot on irritant clay soils, with signs becoming apparent in most patients by the third decade of life. Promoting use of footwear in areas of irritant soil and good foot hygiene are required in extended periods of elevation, and occasionally nodulectomy. |
| Deribe et al., 2020 | To estimate the health and economic burden of podoconiosis in Ethiopia | National | Economic evaluation | Podoconiosis | In 2017, there were 1.5 million cases of podoconiosis in Ethiopia, which corresponds to 172073 disability adjusted life years or 182 per 100000 people. The economic burden of podoconiosis in Ethiopia is estimated to be US$213.2 million annually. The 91.1% of this resulted productivity costs. The average economic burden per podoconiosis case was US$136.9. |
| Masraf et al., 2020 | To compared the age-standardized mortality ratios (SMRs) of two datasets from northern Ethiopia | National | Cohort | Podoconiosis | Podoconiosis patients experience high mortality compared to the general population, with annual crude death rate per 1000 population for podoconiosis patients was 28.7. |
| Bekele et al., 2016 | To assess podoconiosis burden in Ethiopia | Wollega  Ethiopia | Community-based cross-sectional | Podoconiosis | Prevalence of podoconiosis in the population was 3.05%. The prevalence was significantly higher among women (3.67%) than men (2.4%). Most (92.2%) people with podoconiosis were in the economically active age group (15–64 years). |
| Deribie et al., 2020 | To identify a set of clinical features that, combined into an algorithm, allow for diagnosis of podoconiosis | Northern Ethiopia | Cross-sectional | Podoconiosis  (Diagnostics) | clinical algorithm of clinical history and physical examination could be used in areas suspected or endemic for earlier identification of podoconiosis. |
| Caprioli et al.,2020 | To determine the impact on caregivers for patients with leg lymphoedema in a co-endemic district of Ethiopia | SNNP, Ethiopia | Primary cross-sectional | LF and podoconiosis | LF and podoconiosis caused Lymphoedema impacts patients' and their caregivers' lives negatively. Emphasizes the importance of access to effective morbidity management and disability prevention services to reduce the burden help to address the SDG 5, target 5.4. |
| Tekola et al., 2012 | To assess podoconiosis/tropical lymphedema resulting from long-term barefoot exposure to red-clay soil derived from volcanic rock | Southern Ethiopia | Experimental | Podoconiosis | The association between variants in HLA class II loci with podoconiosis (as NCD). T-cell-mediated inflammatory disease and modelling for gene-environment interactions that may be relevant to other complex genetic disorders. |
| Melkie et al.,2020 | To assess the prevalence and associated factors of active trachoma among children aged 1-9 years old in mass drug administration | Northwest Amhara Region, Ethiopia | Comparative cross-sectional | Trachoma | Active trachoma was high in the study area that has a significant variation between graduated and non-graduated districts with MDA. fly presence in the house, having more than two children in a household, did not wash the face daily, not use soap during face washing, and dirt on the child's face were the significant predictors of active trachoma |
| Alambo et al., 2020 | To assess the prevalence of active trachoma and associated factors | South Ethiopia | Community-based cross-sectional | Trachoma | 37.9% of children aged 1-9 years have active trachoma. Households without latrine, openly disposing domestically produced waste, cooking in the same room, using the cooking room without a window and caretakers’ having inadequate knowledge about trachoma were more likely to have their children develop active trachoma. |
| Gebrie et al., 2019 | To determine the prevalence and associated factors of active trachoma among children in Ethiopia | National | Systematic review and meta-analysis | Trachoma | Prevalence of active trachoma among children in Ethiopia was 26.9%. The highest prevalence was reported in SNNP (35.8%), the lowest was reported in Oromia region (20.2%). Absence of latrine, the unclean faces of children, and no reported use of soap for washing have shown positive association with active trachoma among children |
| Sata et al., 2021 | To provide an update on the prevalence of trachoma among children aged 1–9 years as of the most recent impact or surveillance survey in all 160 districts of Amhara | Amhara region, Ethiopia | Longitudinal trend analysis | Trachoma | About 28% of districts had trachomatous inflammation-follicular (TF). A strong commitment to the SAFE strategy coupled with data-driven enhancements to that strategy is necessary to facilitate timely elimination of trachoma as a public health problem regionally in Amhara and nationwide in Ethiopia. |
| Astale et al.2021 | To estimate the population-based prevalence of TS within Amhara, Ethiopia, a region with a historically high burden of trachoma | Amhara, Ethiopia | multi-stage cluster surveys | Trachoma | The prevalence of trachomatous scarring (TS) was 8.2%, and individuals ages 15 years and older was 12.6%. District-level TS prevalence among individuals ages 15 years and older ranged from 0.9 to 36.9 %. |
| Burn et al., 2017 | To explore correlates of podoconiosis with cataract or trachomatous trichiasis among this population | Amhara region | Cross-sectional | Trachoma, podoconiosis | Individuals with podoconiosis have a higher burden of trachomatous trichiasis (TT) and worse visual acuity than their matched healthy neighborhood controls. |
| Last et al., 2020 | To infer an indication of their relative importance to transmission of trachoma | Oromia Ethiopia | Population-based survey | Trachoma  (Diagnostics) | Active trachoma and ocular Chlamydia trachomatis were detected in 10% and 2% of all-ages, and 21% and 3% of 1–9-year-olds, respectively. Chlamydia trachomatous was detected in 12% of tested non-ocular swabs from ocular-positive. |
| Ebert et al., 2019 | To explore factors associated with individual MDA participation at the individual. | Amhara Region  Ethiopia | Community-based cross-sectional | Trachoma  (MDA) | MDA coverage ranged from 78.5% to 86.9%. The MDA campaign and knowledge of trachoma were all positively associated with MDA participation. |
| Leta et al., 2020 | To diagnostic examination strategies during the national mapping of STH and Schistosoma mansoni in Ethiopia | Amhara Region | national mapping | STH & Schistosomiasis (diagnostics) | 70% reduction in time required for sample testing, but reduced total operational costs by only 11% |
| Yimam et al., 2022 | To compute pooled prevalence, estimate of intestinal parasites and associated pooled | National | Systematic review and meta-analysis | Intestinal parasite | Prevalence of IPs among food handlers at food service establishments in Ethiopia was 33.6%. Ascaris lumbricoides (8.8%) were the most predominant IP. |
| Assemie et al., 2021 | To determine prevalence and associated factors of intestinal parasite among primary school children | National | Systematic reviews and meta-analysis | Intestinal parasite | The pooled prevalence for at least one intestinal parasite was 46.09%. Ascaris lumbricoides (13.98%) and hookworm (12.51%) were the top causes of parasitic infection among school children in Ethiopia. |
| Hailu et al., 2021 | To determine the pooled prevalence of S. stercoralis at country, and regional state levels. | National | Systematic review and meta-analysis | Strongyloides stercoralis  (diagnostics) | Prevalence of S. *stercoralis* in Ethiopia was 1.82 %. The prevalence of S. stercoralis was 8.9% in Addis Ababa city. High prevalence of S. *stercoralis* was found to be 44.02 % with a combination of formol ether concentration, Baermann concentration, and molecular methods. Low prevalence of 0.26 %, 0.31 %, and 1.20 % was evidenced respectively with Kato-Katz, direct saline microscopy, and formol ether concentration methods. |
| Terefe et al., 2019 | To identified risk factors, diagnosis, prevalence and/or clinical outcomes of strongyloidiasis in Ethiopia | National | Systematic review and meta-analysis | Strongyloidiasis stercoralis  (Diagnostics) | S. *stercoralis* is an overlooked NTD in Ethiopia that need for a systematic approach of diagnosis using a combination of molecular and serology based diagnostic methods to determine the burden of S. *stercoralis*. |
| Alemu et al., 2020 | To provide conclusive evidence on the intestinal parasite-tuberculosis co-infection in Ethiopia. | National | Systematic review and meta-analysis | Intestinal parasite | Prevalence of IPs co-infection was 33%, and the most common IPs were Ascaris *lumbricoides* 10.5%, Hookworm 9.5%, and S. *sterocoralis* 5.6%. |
| Eshetu et al., 2020 | Assess efficacy of single (500 mg) versus multiple doses (100 mg twice a day during three consecutive days) of mebendazole against hookworm | Northwest Ethiopia | Randomized clinical trial | STH (hookworm) | The single dose regimen of mebendazole for the treatment of hookworm infections showed poor cure and egg reduction rates, while the multiple doses revealed satisfactory. |
| Woldeyohannes et al., 2021 | To determine the pooled prevalence of Schistosomiasis and its association with gender of school age children in Ethiopia. | National | Systematic review and meta-analysis | Schistosomiasis (S. *mansoni* and S. *haematobium*) | Prevalence of schistosomiasis in Ethiopia was 28.8%, in which schistosomiasis ranged from 14.95% in Afar to 39.77% in SNNP. Male children were 58% more likely infected with Schistosomiasis than female children. |
| Shumbej et al., 2019 | To determine the impact of annual preventive mass chemotherapy for soil-transmitted helminths among schoolchildren in an endemic area of | Gurage, Ethiopia | Cross-sectional | STH (MDA) | Although annual mass chemotherapy failed to clear STHs that resulted in a substantial reduction in the burden and infection intensity. Therefore, deworming for school children and access to improved WASH in school should be emphasized to interrupt transmission. |
| Bisetegn et al., 2021 | To indicate the prevalence of Schistosoma mansoni among children at the national and regional levels | National | Systematic review and meta-analysis | Schistosomiasis mansoni | The prevalence of schistosoma mansoni infection among children was 37.1%. This is an alert to improve and implement appropriate control strategies such as MDA in Ethiopia. |
| Ponpetch et al., 2021 | To review S. *mansoni* prevalence of infections and describe key biogeographical characteristics in the endemic areas in Ethiopia. | National | Systematic review and meta-analysis | Schistosomiasis mansoni | Prevalence of Schistosomiasis mansoni ranged from 0.5% to 99.5%. 36.8% of the survey sites had adjusted prevalence of infection higher than 50%. S. *mansoni* endemic areas were distributed in six regional states with the majority of surveys being in Amhara and Oromia. |
| Abdella et al., 2021 | To describe the clinical profile and management of Hepatosplenic schistosomiasis in one of Ethiopia’s referral hospitals | National | Hospital-based cross-sectional | Hepatosplenic schistosomiasis (diagnostic) | Most patients (70%) presented with upper gastrointestinal-transfusion. Hepatitis B coinfection was documented in 11 patients (20%). Upper gastrointestinal endoscopy was performed in 31 patients (56%). Praziquantel was administered to 11 patients (20%). |
| Assefa, 2018 | To summarize and pool estimates of studies that report the prevalence of leishmaniasis in Ethiopia | National | Systematic review | Leishmaniasis (diagnostic) | Prevalence of leishmaniasis was 19%. Diagnosis method used have contributed to the heterogeneity of studies. Molecular diagnosis has significantly lower prevalence than microscopic examination. |
| Haftom et al., 2020 | To determine the prevalence and risk factors of human leishmaniasis in Ethiopia | National | Systematic review and meta-analysis | Leishmaniasis | Prevalence of leishmaniasis was 9.13%, and it ranged from 2.3% in Tigray to 39.1% in Amhara region. The significant risk factors being male and presence of hyraxes within a 300-m radius of the sleeping area. |
| Gebreyohannes et al.2018 | To obtain stronger evidence on treatment outcomes of VL from the existing literature in Ethiopia | National | Systematic review and meta-analysis | V. leishmaniasis  (Access to medicine) | The overall treatment success rate was 82.6%. For patients treated with sodium stibogluconate (SSG), success rates at the end of treatment and at six-month follow-up were 81.5% and 80.7%, respectively. Multiple doses of liposomal-amphotericin B (L-AMB) had treatment success rates of 96.7% and 71–100% at the end of treatment and at 6 months follow-up, respectively. The combination of SSG with paromomycin (PM) gave treatment success rates of up to 90.1% at the end of treatment. |
| Merdekios et al, 2021 | To compare microscopy with five different molecular methods on two different sample types collected from skin lesions of suspected CL patients in the south of Ethiopia | Southern Ethiopia | Cross-sectional | Leishmaniasis  (Diagnostics) | Sensitivity of microscopy, ITS PCR, SSU PCR, Mary kDNA PCR, LC kDNA PCR and SL RNA PCR were respectively 52%, 22%, 64%, 99%, 100% and 94%. The kDNA PCRs showed excellent performance for diagnosis of L. aethiopica on SS. Lower-cost SL RNA detection can be a complementary high-throughput tool. |
| Abbasi et al., 2013 | To elucidate the role of symptomatic and asymptomatic Leishmania donovani infected persons in the epidemiology of VL in Northern Ethiopia | Northern Ethiopia | Cross-sectional | Leishmaniasis  (Diagnostics) | Although qRT-kDNA PCR is a highly sensitive test, the dependability of low positives remains questionable. While optimal sensitivity is achieved by targeting k-DNA. It is important to validate the causative species of VL by DNA sequencing. |
| Azene et al., 2020 | To estimates the overall prevalence of scabies and associated factors in all age groups in Ethiopia | National | Systematic review and meta-analysis | Scabies | The prevalence of scabies infestation was 14.5% in Ethiopia. Persons from high family size and any contact with scabies case were factors associated with scabies. |
| Worku et al., 2020 | To identify the determinants of scabies outbreak in Takusa district, Northwest Ethiopia | Ethiopia | Community-based case-control study | Scabies | Frequent contact with people who had scabies, not using detergents/soap for washing, and mobility of people from non-epidemic to the epidemic areas were determinants. |
| Wochebo et al.,2019 | To investigate scabies outbreak, identify risk factors, and recommend preventive measures in Kechabira district, Kembata Tembaro zone, Southern Ethiopia | Southern Ethiopia | Cross sectional | Scabies | The prevalence of scabies was 2.5%. Of these, half (51.9%) of the cases were males and 48.1% were females. The highest cases were seen in children aged 5-14 (50.6%) years. Sharing clothes with scabies patients, and households having greater than six family members were the identified risk factors to scabies outbreak. |
| Azene et al.,2020 | To estimates the overall prevalence of scabies and associated factors in all age groups in Ethiopia | National | Systematic review and meta-analysis | Scabies | The overall prevalence of scabies was 14.5% in Ethiopia. Of these, the highest prevalence was 19.6% in Amhara region. A person from a large family size, and sharing a bed were significantly associated with scabies. |
| Walker et al., 2017 | To determine the prevalence and impact of scabies in schoolchildren in southern Ethiopia. | Southern Ethiopia | Cross-sectional | Scabies | Scabies appeared to have a significant negative effect on quality of life, and the burden of skin disease was 40% having an ectodermal parasitic skin disease. |
| Dagne et al., 2019 | To assess the prevalence of scabies and associated factors among students in primary schools in Dabat district, northwest Ethiopia | Dabat district, northwest Ethiopia | Cross-sectional | Scabies | The prevalence of scabies was 9.3% Being rural school, had illiterate father, being grade level 1-4, rarely taking a bath, contact with a person with itching symptom, a family member with itchy symptoms, not living with both parents, and using water only for hand washing were factors associated with scabies infestation among schoolchildren. |
| Yassin et al., 2017 | To investigate scabies outbreak, identify factors associated with scabies transmission and to take public health action among affected group of people | Amhara, Ethiopia | Matched case-control | Scabies | People who share close from ill person were 2.76 times more likely to develop scabies; and individuals who had close contact with ill person were 5 times more likely to develop disease scabies, also individuals who had travelling history to scabies epidemic area were 4.7 times more likely develop the disease. individuals who wash their body in more than a week interval were 3.22 times more likely develop scabies. |
| Arega et al., 2020 | To assess orphans suffered from scabies, other NTDs and malnutrition in Ethiopia | National | Record review | Scabies | The prevalence of scabies among orphan children was 23.1%. of these, 28.1% of them had multiple episodes. The median age (interquartile range) of the children with scabies was 3 (2-5) months. 85.2% of the orphans with scabies were stunted and /or wasted. |
| Enbiale et al., 2020 | To describe its implementation and report on a) numbers screened and identified with scabies, b) treatment category and drug type and c) human resources used, duration, and cost of the campaign | Northwestern Ethiopia | Cross-sectional | Scabies | Scabies cases was 9.7% and 11.0% of the contacts received treatment. Scabies prevalence varied from 39.2% in Central Gondar, South Gondar (16.7%) and North Gondar (15.0%). Of 93% who received treatment, 94% received Ivermectin, the rest topical permethrin and sulfur. The average coverage capacity of an MDA campaign staff member was 84 people per day. The total cost was 11,696,333 United States Dollars (USD). Cost per 100,000 population = 129,135 USD. |
| Meribo et al., 2017 | To review of Ethiopian Onchocerciasis Elimination Programme | National | Review | Onchocerciasis | MDA delivered two times per year, and has successfully scaled up interventions and achieved 100% geographic coverage in all known endemic districts. The treatment coverage for the last five years has been maintained at more than 80%. Despite many years of ivermectin MDA the transmission of onchocerciasis is still exist in many districts |
| Endeshaw et al., 2015 | To determine the prevalence of LF microfilaremia in onchocerciasis endemic districts that had received 7 years of MDA with ivermectin | North western Ethiopia | Cohort | onchocerciasis  (MDA access) | The mean microfilaremia and density were 4.7%, and 9.8 mf/60 μl, respectively. Children (2–9-year-old) were infected during the MDA period. MDA coverage was 66.4% over a 7-year period, but ivermectin only MDA for onchocerciasis did not interrupt LF transmission. So, albendazole should be added and treatment coverage improved. |
| Diro et al.,2017 | To determine whether peripheral blood could be used instead of invasive tissue aspirates to diagnose VL, using three parasite concentration techniques | North western Ethiopia | Cross-sectional | V. Leishmaniasis  (Diagnostics) | VL could be ruled-in with peripheral blood microscopy in a substantial number of VL suspect among HIV-coinfected patients. More sensitive and logistically feasible methods than light microscopy is needed to detect Leishmania *donovani* parasites present in blood |
| Kiros & Regassa, 2017 | To determine the sensitivity and specificity of rk39-ICT in the diagnosis of VL in Ethiopia | Northern Ethiopia | Cross-sectional | visceral leishmaniasis (diagnositics) | Sensitivity of rK39-ICT is low and its specificity is poor. Significant number of patients with confirmed VL, did not have travel history to endemic areas. The rK39-ICT needs improvement for clinical use in our set up and case definition for visceral leishmaniasis in Ethiopia. |
| Aberra et al. 201 | To produce and evaluate the performance of an in-house liquid (AQ) direct agglutination test (DAT) antigen | National | Cross-sectional | Leishmaniasis (Diagnostics) | Although further standardization is required, the in-house AQ-DAT could improve diagnostic accessibility, minimize intermittent stock outs and strengthen the national VL control program |
| Griensven et al., 2016 | To assess treatment outcomes of CL due to L aethiopica in order to help identify potentially efficacious medications on CL | National | Systematic review (Access to medicine) | Cutaneous Leishmaniasis | antimonials, pentamidine and cryotherapy were the most commonly used drugs. With cryotherapy, cure rates were 60–80%, and 69–85% with antimonials. Pentamidine appeared effective against complicated CL, also in cases non-responsive to antimonials. |
| Diro et al., 2019 | To assess acceptable efficacy a combination of a lower dose of AmBisome with miltefosine treatment. | National | Randomized trial (access to medicine) | visceral leishmaniasis | The AmBisome adjusted efficacy of was 55% in the monotherapy arm, and 88% in the combination arm. |
| Surur et al., 2020 | To identify challenges and opportunities for drug discovery for C. Leishmaniasis in developing countries | Ethiopia | Review | C. Leishmaniasis | The current paradigm of drug development for NTDs has failed miserably. A new model focusing on building partnerships and capacity in developing countries has to be prioritized. |
| Abongomera et al., 2020 | To identify prognostic factors for mortality among V. Leishmaniasis patients | Ethiopia included | Systematic review and meta-analysis | visceral leishmaniasis | Twelve prognostic factors were evaluated in five or more studies and these results were submitted to meta-analysis producing one pooled crude odds ratio per prognostic factor. |
| Coulborn et al., 2018 | To assess barriers, and recommend interventions to increase access, to VL diagnosis and care | Northern Ethiopia | Qualtative study | Leishmaniasis | very poor access to diagnosis and, consequently, significantly delayed access to treatment. |
| Aberra et al. 2019 | To evaluate the diagnostic performance of MCM for the diagnosis of CL caused by L. aethiopica | Ethiopia | Cross-sectional | Cutaneous leishmaniasis  Diagnostics) | microculture method (MCM) is a sensitive and a rapid culturing method for the isolation of L. aethiopica than traditional culture method (TCM) and smear microscopy |
| Adugna et al., 2017 | To compare the performance of Mini Parasep® solvent-free (SF) faecal parasite concentrator, Kato-Katz thick smear and McMaster techniques | Southern Ethiopia | Cross-sectional | Intestinal parasite  (Diagnostics) | The Mini Parasep® SF faecal parasite concentrator technique showed better performance than the Kato-Katz and McMaster techniques for the detection of intestinal helminth infections in stool samples, particularly for S. *mansoni*, A. *lumbricoides* and H*. nana*. |
| Hoffman et al., 2019 | To assess sensitivity and specificity of 3D and 2D photography as a tool for training TG to detect TT | National | Cross-sectional study | trachomatous trichiasis  (Diagnostics) | The slightly higher sensitivity of 2D photos comes at considerable cost in specificity. 3D was preferred to conventional 2D photos for training. Standardized 3D images of TT could be a useful tool for training TG. |
| Stoller et al., 2020 | To assess an emergence of infection with ocular Chlamydia trachomatis after mass treatment with antibiotics | National | Cluster randomized trial | Trachoma  (Access to medicine) | Single dose of oral Azithromycin or topical Tetracycline were the common treatment. Through intensive latrine promotion, trachoma was reduced from 45.5% and 43.0%, respectively. Latrine and non-latrine household latrine coverage and use were 80.8% and 61.7%, respectively. |
| Keenan et al., 2028 | To assess the effect of continuation versus discontinuation of mass azithromycin treatment | National | Cluster randomized trial | Trachoma  (access to medicine) | Ocular chlamydia infection rebounded after 4 years of periodic mass azithromycin distribution, and continued distributions did not completely eliminate infection in all communities or meet WHO control goals. |
| Adafrie et al., 2021 | To assess the uptake of trachoma trichiasis (TT) surgery in Southern Tigray, Ethiopia | Northern Ethiopia | Mixed method cross-sectional | Trachoma  (Access to medicine) | 57.9% of patients utilized TT surgery. Absence of someone to care the family, companion, nearby health facility, work load, fear, and believing eye drop can treat TT were significantly associated factors. |
| Oldenburg et al., 2019 | To conduct a pooled analysis of all published cluster-randomized trials evaluating the effect of azithromycin MDA on child mortality | Ethiopia was part of the study | Pooled analysis RCT Data | Trachoma | There was a 14.4% reduction in all-cause child mortality in com- munities receiving azithromycin MDA. Mortality rate was 15.9 per 1,000 person-years, but mortality rate was lower in azithromycin-treated communities than in placebo-treated communities 14.7 deaths per 1,000 person-years versus 17.2 deaths per 1,000 person-years. |
| Bayissasse et al. 2020 | To determine whether M. *sorbens* females were attracted to volatile odours from human faeces in preference to odours from the faeces of other animals, | Gambia and one in Ethiopia | Cross-sectional | Trachoma | Twelve compounds are putative attractants that may play a role in this response, by identifying, for the first time, compounds including short chain fatty acids and aromatic compounds that are detected by the antennae of M. *sorbens*. Further work is required to optimise chemical blends and release rates, to produce a synthetic lure to which the behavioral responses of M. *sorbens* can be investigated. |
| Mulugeta et al., 2018 | To assess mass Zithromax administration coverage, social mobilization and campaign challenges | Tigray, Ethiopia | Cross-sectional | Trachoma | The coverage of Zithromax MDA was 93.3% which is higher than the minimum WHO set criteria of 80%. The MDA coverage was lower in urban than rural. Misconceptions and poor mobilization were common challenges. |
| Habtamu et al., 2018 | To assess doxycycline might reduce the risk of postoperative trichiasis following surgery in patients with trachomatous trichiasis | National | RCT | Trachoma  (Access to medicine) | Doxycycline did not reduce the risk of postoperative trichiasis and is therefore not indicated for the improvement of outcomes following TT surgery. Surgical programmes should continue to make efforts to strengthen surgical training and supervision to improve outcomes |
| Asfaw et al., 2021 | To assess equity and coverage in MDA and identifying factors associated with drug coverage for STH infections among SAC in the hard-to-reach setting of southern Ethiopia | Southern Ethiopia | Community -based cross-sectional | STH  (Access to medicine) | The overall MDA coverage for STH was found to be 27.5%. Having taken drugs were highest among school-enrolled children and in those who knew the purpose of MDA. Drug uptake was lower by 69% among those who had got informed only when the drugs delivered and by 92% among those who needed to travel >30 minutes to reach drug-distribution |
| Shahvisi et al., 2018 | To identify the rights violations is key to characterizing the scale and nature of the problem, identifying duties is critical to eliminating podoconiosis | National | Review | Podoconiosis  (Access to care) | describe the duties of the Ethiopian government, the international community, and those sourcing Ethiopian agricultural products in relation to promoting shoe-wearing, providing adequate health care, and improving health literacy |
| Noordende et al., 2016 | pilot a family-based intervention to support prevention and self- management of leprosy, LF and podoconiosis-related disabilities in Ethiopia |  | quasi-experimental pre/post mixed methods | leprosy, LF, and podoconiosis | The family-based intervention had a positive  effect on impairments and self-management of disabilities, family quality of life and stigma. Validated measurement tools, determine its effectiveness and long-term sustainability are needed action. |
| Phillips et al., 2018 | To explore the experiences and impact of acute attacks on the caregivers of those with podoconiosis in one endemic district of Ethiopia | Oromia, Ethiopia | Qualitative study | Podoconiosis | Significant social and financial pressures placed on podoconiosis- affected families which are exacerbated during acute attacks. This study also highlighted the emotional burden experienced by caregivers, the range of care activities placed on them and the limited support available. |
| Tora et al., 2017 | To explore the health beliefs of school-age rural children in podoconiosis-affected families. | Southern Ethiopia | Qualitative study | Podoconiosis | Overcoming practical challenges such as shortage of footwear and other hygiene facilities requires and livelihood strengthening activities are need for linking podoconiosis-affected families with local governmental or NGOs in providing socio-economic support for households. |
| Tsegay et al., 2015 | To explore barriers to access and re-attendance of patients with podoconiosis in northern Ethiopia | Northern Ethiopia | Qualitative study | Podoconiosis | Barriers to access and to continued attendance at treatment centers were lay beliefs about the disease’s causation and presentation, occupational, geographic and financial barriers, stigma and conflicting expectations of treatment services |
| Ayode et al., 2013 | To understand the behaviors that influence use of footwear will lead to improved ability to measure shoe use | Southern Ethiopia | Qualtative study | Podoconiosis | Identified several barriers to shoe wearing that are amenable to intervention and which we anticipate will be of benefit to those considering NTD prevention through shoe distribution |
| Wharton-Smith et al., 2019 | To address this gap by applying a gender lens to health seeking  for five NTDs | SNNP, Ethiopia | Qualitative study | Podoconiosis | Gender related factors affected care seeking for NTDs and were described as reasons for not seeking care, delayed care seeking and treating NTDs with natural remedies. Women faced additional challenges in seeking health care due to gender inequalities. |
| Teshome et al., 2021 | To assess the difference between the reported coverage and actual coverage of Ivermectin (IVM) and Albendazole (ALB) treatment given for LF | Gambella  Ethiopia | Cross-sectional | Lymphatic filariasis | Coverage for LF treatment was 81.5%. They were offered the treatment 823 (99.6%) swallowed the drug. The coverage in school age children (5–14) shows significant difference with treatment coverage in individuals aged 15 and above in the last MDA campaign. |
| Engdawork et al., 2020 | To measure stigmatizing attitudes and associated risk factors among rural youth in southern Ethiopia | Southern Ethiopia | Cross-sectional | Podoconiosis  (access to care) | 52.7% of the youth who held more stigmatizing attitudes toward patients with podoconiosis, 59.3% were females and 96.6% did not have affected friends. |
| Sime et al., 2018 | Ethiopian experience of implementing the new confirmatory mapping tool and discuss the implications of the results for the LF program in Ethiopia | National | Mapping of tool | Lymphatic filariasis endemicity | new confirmatory mapping tool for LF can benefit national LF programs by generating information that not only can confirm where LF is endemic, but also can save time and resources by preventing MDA where there is no evidence of ongoing LF transmission |
| Negussie et al., 2018 | To test the hypothesis that a simple lymphoedema treatment package would reduce the incidence of acute. | Northwestern Ethiopia | pragmatic randomised controlled trial | Podoconiosis  (Access to medicine) | A simple, inexpensive package of lymphoedema self-care is effective in reducing the frequency and duration of acute dermatolymphangioadenitis. |
| Samuel et al., 2016 | to evaluate the impact of six years CDTI on parasitological and clinical indices of Onchocerciasis | Western Ethiopia | Cross-sectional | Onchocerciasis (Access to medicine) | Community directed treatment with Ivermectin in the Control of Onchocerciasis in Ethiopia |
| Churko et al., 2021 | To validate onchocerciasis treatment coverage in the selected districts of Ethiopia. | Two districts, Ethiopia | Cross-sectional | Onchocerciasis | The overall treatment coverage of onchocerciasis in the two selected districts of Ethiopia was 85.9% of the eligible population. School attendance was also significantly associated with treatment offering and swallowing status. |
| Dana et al., 2020 | To determine the diagnostic performance of DWMM among school children in Jimma Town, South-West Ethiopia | Jimma, Ethiopia | Cross-sectional | Diagnostic  STH | STH diagnostic method was diagnostic sensitivity of DWMM that was compared to a composite reference standard consisting of Kato-Katz, McMaster and Mini- FLOTAC. The sensitivity of DWMM was 73.8% for Ascaris, but was around 17% for both Trichuris and hookworms. |
| Tesfie et al., 2020 | To evaluate the effectiveness of praziquantel for S. *mansoni* treatment. | Northwestern Ethiopia | Cross-sectional | Schistosomiasis (Access to medicine) | S. *mansoni* prevalence was high. Praziquantel is an effective drug for the treatment of S. mansoni. This high prevalence of S. *mansoni* requires MDA of praziquantel |
| Levecke et al., 2020 | To assess the therapeutic efficacy of a single oral dose of PZQ (40 mg/kg) against Schistosoma mansoni | Ethiopia | Multicounty trial study | Schistosomes  (access to medicine) | Evaluating PZQ efficacy as described by the WHO. Overall, therapeutic efficacy, measured as the reduction in arithmetic mean of schistosome egg counts following drug administration (S. *mansoni*: 93.4%); S*. haematobium*: 97.7% and S. *japonicum*: 90.0%). |
| Cools et al., 2019 | To evaluate the diagnostic performance of different diagnostic methods for the detection and quantification of STHs in stool | Ethiopia was part of the study | Cross-sectional | STH  (Diagnostics) | The diagnostic performance of a single Kato-Katz is underestimated by the community and that diagnostic specific thresholds to classify intensity of infection are warranted for Mini-FLOTAC, FECPAKG2 and qPCR. |
| Chisha et al., 2020 | To quantify national and district disaggregated treatment coverage status for SCH and compare validated coverage with the one reported | National | Cross-sectional | Schistosomiases  (Access to medicine) | Over all treatment coverage of PZQ against schistosomiasis was 75.5%. Although it is in accordance with WHO recommendation for Ethiopia, national programmatic improvements are necessary to achieve higher coverage in the future. |
| Hounsome et al., 2020 | To integrate a holistic package of physical health, mental health and psychosocial care for podoconiosis, LF and leprosy | Northwestern Ethiopia | Implementation research | podoconiosis, LF and leprosy | Integration of the care package into routine healthcare in Ethiopia may be effective in improving health-related quality of life and disability and reducing time out of economic activity due to illness |
| Kebede et al., 2018 | To examine the clinical burden of lymphoedema and hydrocoele in 20 co-endemic woredas (districts) of Ethiopia. | Ethiopia | Mapping | LF and podoconiosis | Integrated clinical case mapping of both LF and podoconiosis in Ethiopia. This key clinical information will assist and guide the allocation of resources to where they are needed most. |
